# Supplementary material for: Adverse events associated with oseltamivir and baloxavir marboxil in against influenza virus therapy: A pharmacovigilance study using the FAERS database
Source: PLoS One. 2024 Nov 13;19(11):e0308998. doi: 10.1371/journal.pone.0308998 (PMC11560017; doi:10.1371/journal.pone.0308998)
Supplement: Attachment — Submitted filename: response to reviewers1.docx [file pone.0308998.s002.docx]

Dear Reviewer 3,

Thank you for providing valuable comments and suggestions during the review of our manuscript. We greatly appreciate your professionalism and patience with our research work. Regarding the issue you raised, "Higher values of ROR and PRR do not indicate a stronger statistical relationship between the target drug and the target adverse event. This is a misconception by the authors." we regretfully acknowledge our methodological misunderstanding.

Since the FAERS database is a spontaneous reporting system that only includes adverse event cases caused by drug use, it does not contain the number of drug users; therefore, it is impossible to calculate the incidence of adverse events. Disproportionality analysis signals are necessary for identifying unknown adverse events.

In the article you sent, the limitations of these disproportionality algorithms were mentioned, as well as how many studies use certain terms that lead to misleading causal conclusions. This is inappropriate, and we have revised the terminology in our article according to the suggestions from your paper to provide readers with more accurate conclusions. Additionally, we have updated the limitations section of our discussion accordingly."Since the FAERS database is a spontaneous reporting system that only includes adverse event cases caused by drug use, it does not contain the number of drug users; therefore, it is impossible to calculate the incidence of adverse events. Disproportionality analysis signals are necessary for identifying unknown adverse events. However, current methods of disproportionality analysis have limitations. For instance, PRR has low specificity, leading to false positive signals, which may require additional classification criteria. On the other hand, BCPNN has low sensitivity, resulting in false negative associations. Thus, the development of more accurate disproportionality analysis methods is crucial[1, 2]. Moreover, many studies exploring drug databases have used disproportionality analysis to draw causal conclusions. It is important to note that disproportionality analysis does not allow for establishing any causal inferences. However, recent reports indicate that claims of causality based on disproportionality analysis results are not uncommon. Therefore, more reasonable interpretations of relationships derived from pharmacovigilance analysis are essential[3, 4]".

1.	Noguchi Y, Tachi T, Teramachi H. Detection algorithms and attentive points of safety signal using spontaneous reporting systems as a clinical data source. Brief Bioinform. 2021;22(6). Epub 2021/08/29. doi: 10.1093/bib/bbab347. PubMed PMID: 34453158.

2.	Noguchi Y, Yoshizawa S, Aoyama K, Kubo S, Tachi T, Teramachi H. Verification of the "Upward Variation in the Reporting Odds Ratio Scores" to Detect the Signals of Drug-Drug Interactions. Pharmaceutics. 2021;13(10). Epub 2021/10/24. doi: 10.3390/pharmaceutics13101531. PubMed PMID: 34683823; PubMed Central PMCID: PMCPMC8537362.

3.	Fusaroli M, Salvo F, Begaud B, AlShammari TM, Bate A, Battini V, et al. The REporting of A Disproportionality Analysis for DrUg Safety Signal Detection Using Individual Case Safety Reports in PharmacoVigilance (READUS-PV): Explanation and Elaboration. Drug Saf. 2024;47(6):585-99. Epub 2024/05/07. doi: 10.1007/s40264-024-01423-7. PubMed PMID: 38713347;

4.	Mouffak A, Lepelley M, Revol B, Bernardeau C, Salvo F, Pariente A, et al. High prevalence of spin was found in pharmacovigilance studies using disproportionality analyses to detect safety signals: a meta-epidemiological study. J Clin Epidemiol. 2021;138:73-9. Epub 2021/06/30. doi: 10.1016/j.jclinepi.2021.06.022. PubMed PMID: 34186195.
